# Effects of native pollinator communities on the physiological and chemical parameters of loquat tree (*Eriobotrya japonica*) under open field condition

**DOI:** 10.1016/j.sjbs.2021.02.062

**Published:** 2021-02-27

**Authors:** Saboor Ahmad, Ahlam Khalofah, Shahmshad Ahmed Khan, Khalid Ali Khan, Muhammad Jawad Jilani, Taimoor Hussain, Milan Skalicky, Hamed A. Ghramh, Zubair Ahmad

**Affiliations:** a Institute of Apicultural Research/Key Laboratory of Pollinating Insect Biology, Ministry of Agriculture, Chinese Academy of Agricultural Sciences, Beijing 100093, China; b Department of Entomology, Faculty of Crop and Food Sciences, Pir Mehr Ali Shah (PMAS) Arid Agriculture University Rawalpindi, 46000, Pakistan; c Biology Department, Faculty of Science, King Khalid University, P.O. Box 9004, Abha 61413, Saudi Arabia; d Research Centre for Advance Material Science (RCAMS), King Khalid University, P.O. Box 9004, Abha 61413, Saudi Arabia; e Unit of Bee Research and Honey Production Faculty of Science, King Khalid University, P.O. Box 9004, Abha 61413, Saudi Arabia; f Centre for Integrative Ecology, School of Life and Environmental Sciences, Melbourne Burwood Campus Deakin University, Australia; g Department of Agronomy, Faculty of Crop and Food Sciences, Pir Mehr Ali Shah (PMAS) Arid Agriculture University Rawalpindi, 46000, Pakistan; h Department of Botany and Plant Physiology, Faculty of Agrobiology, Food and Natural Resources, Czech University of Life Sciences Prague, Kamycka 129, 165 00 Prague, Czechia; i Biology Department, Faculty of Sciences and Arts, King Khalid University, Dhahran Al Janoub, Saudi Arabia

**Keywords:** Pollinators diversity, Hymenopteran, *Eriobotrya japonica*, Abundance, Fruit yield, Physiological parameters

## Abstract

Wild and managed pollinators are the key component of biodiversity, contributing to important ecosystem services such as pollination and supporting human food security. Pollination by insects is a crucial component of the food chain that ensures the production of fruits and strongly affects the fruit quality, but the effect of insect pollination on fruit physiological and chemical parameters is largely unknown. The current study was conducted to determine the insect pollinators diversity and their relative abundance in the loquat (*Eriobotrya japonica*) orchard during 2017–2019. Further, the effect of insect pollinators pollination on the physiological and chemical parameters of fruit quality as compared to control pollinated flowers was investigated. The results revealed that a total of 22 species from 3 families (Apidae, Halictidae, and Syrphidae) were identified during the flowering season. The Apidae and Syrphidae were the most frequently observed families with major groups honey bees (67.89%) and hoverflies (21.57%), respectively. Moreover, results indicated that the fruit yield by the open-pollinated flowers (22.31 ± 0.34 kg/tree) was significantly higher than the control pollinated flowers (14.80 ± 0.25 kg/tree). Physiological and chemical parameters of loquat fruit differed significantly when fruits obtained from open-pollinated flowers as compared to control pollinated flowers. These results suggested that native insect pollinators play important role in the fruit quality of loquat. Hence, maintenance of appropriate habitat of native pollinators near loquat orchards is necessary to ensure good productivity and fruit quality.

## 1 Introduction

Pollination is a very important process in the maintenance of naturally healthy and biodiverse ecosystems (Ollerton et al., 2011). Plants diversity and abundance rely mainly on their interaction with pollinating animals, particularly insects (Abrol, 2011). Insects play a critical role to provide pollination services globally, mainly bees and some flies (hoverflies) that are managed or wild also provide an ecosystem service to various crops (Potts et al., 2016). Pollinators provide several benefits to human especially securing diverse seed and fruit supply, sustaining population of plant diversity, and supporting other cultural values. The estimated value of pollination ranges between 153 and 167 US dollars across the world (Majewski, 2018). Almost 35% of the crop production comes from the species that directly benefit from insect pollination (Klein et al., 2007). The majority of studies have been conducted to examine the effects of floral traits including floral scent, color, nectar, flower number, corolla size, and fruit set on pollinator attraction (Conner and Rush, 1996, Latif et al., 2019b, Hakami et al., 2020, Karar et al., 2020). For instance, it was a documented that increased flower number and size causes increased visits by hoverflies (Conner and Rush, 1996, Caruso et al., 2019). Whereas decreased insect pollination leads to the reduction of flowers, seed set, and yield. Pollinator-depended crops are the main source of various micronutrients including calcium, fluoride, vitamins (A and C), and folic acid (Smith et al., 2015).

Loquat (*Eriobotrya japonica* Lindl.) is an evergreen medium-sized fruit tree that starts flowering in winter (Freihat et al., 2008). It is a rosaceous fruit tree that is native to south-eastern China (Zhang et al., 1990), and cultivated successfully in subtropical, the Mediterranean, and temperate climates of the world (Crane and Caldeira, 2006, Sharpe, 2010, Ercisli et al., 2012). The loquat flowers are small white or yellowish, bowel shaped, and appear in late winters and fruits are ripening during March and April, when no other fruit is available in the market (Hussain et al., 2011). Flowers appear on panicles of 30 to 100 in numbers and fruits also appear in clusters (Hussain et al., 2011). Loquat plant has ornamental significance and is grown for its use in the medicinal application for the treatment of lung-related diseases asthma, cough, and chronic bronchitis (Zhang et al., 2015, Yang et al., 2018). *E. japonica* is not considered as the cross-pollinated fruit in contrast to the apple, pear, and almond (Cuevas et al., 2003, Garratt et al., 2014). As the flowers are available during the scarce period and the loquat (*E. japonica*) flowers have high nectar and pollen and attractive to the wide variety of pollinators (McGregor, 1976, Merino and Nogueras, 2003). The loquat tree is pollinated by the diversity of insect pollinators including honey bees, hoverflies, houseflies, cabbage white butterflies Bombinae, and Myrmeleontidae (Crane and Caldeira, 2006). Different studies prove that the different pollination treatment types not only affect the production quantity but also the quality of the fruits and vegetables (Cuevas et al., 2003, Freihat et al., 2008, Bashir et al., 2018, Latif et al., 2019a, Shakeel et al., 2019, Ali et al., 2020). For instance, pollination improves the quality parameters such as sugar and acid contents in ornamental melon (Shin et al., 2007), reduces the empty seeds in buckwheat (Bartomeus et al., 2014) and, high oil with fewer chlorophyll contents in oilseed rape (Bommarco et al., 2012, Bartomeus et al., 2014). Pollination also improves the commercial-grade of strawberries (Bartomeus et al., 2014), and improve the shape, size, and commercial grade of apples (Garratt et al., 2014), improve the fruit set and fruit quality (shape, size, and weight) and total soluble solid contents of loquat (Cuevas et al., 2000, Cuevas et al., 2003). To better understand the relationships between pollination service and fruit productivity, it is critical to quantify correctly how changing pollinator diversity will affect food quality and production. Data on pollinators diversity of loquat is very limited.

The present study was conducted to determine the insect pollinators diversity and their abundance in the loquat orchard in the Pothwar region of Pakistan. We further investigated the physiological and chemical changes that occurred in fruit quality that is obtained from open-pollinated flowers in comparison to control pollinated flowers. Managing the optimum level of insect pollinators in loquat orchard is essential to improve the fruit quality and income of local loquat growing farmers.

## 2 Materials and methods

### 2.1 Experimental site

The present study was designed to determine the status of pollinators in loquat orchards at district Chakwal (32°55′49″N 72°51′20″E) Punjab, Pakistan. This study was conducted between February 2017 to March 2019. In Pakistan, loquat is considered as minor fruit and covers about 1501 ha with a production of 13,159 tons of which more than 98% comes from the Punjab and Khyber Pakhtunkhwa (Hussain et al., 2009).

### 2.2 Diversity and relative abundance of insect pollinators

The insects visit to the loquat plants were observed weekly over a period of 12 weeks during the months of February and March 2017 to 2019 consecutively. The insect pollinators were categorized as broad taxonomic groups such as honey bees, bumblebees, orchard bees, hoverflies, and ‘other bee’ insects. The abundances of insect pollinator visits were recorded for 10 min for randomly selected 5 plants between 10 AM and 4 PM on sunny days at each seven observation sites. If the loquat plants did not have any flowers, visitors were not recorded. The collected insect pollinators were killed and were subsequently identified by using the available literature.

### 2.3 Fruit set and yield measurement

In each site, random trees were selected to determine the efficacy of open and control pollination on loquat fruit set and fruit quality. For open pollination, a total of 35 trees and 140 branches were selected for the present study. In each site, one loquat tree was chosen as the control pollination. The whole tree was covered with mosquito nets especially prepared for this experiment. After covering the control trees, one box of *Apis mellifera* was placed inside the nets during the blooming periods to ensure good pollination. For the control group, a total of 7 trees and 28 branches were observed to assess the fruit set and fruit quality. To measure the yield (kg) of (open and control pollination selected trees), the total number of fruits were harvested and weighed using an electric balance (Mettler Toledo, Colombus, USA). Fruit harvesting was done on the same day at full ripening for all trees selected for the study. Additionally, fruit samples were taken for analyses of physiological and chemical parameters.

### 2.4 Fruit physical properties

Physical properties of loquat fruit including fruit weight (g), size (mm), diameter, number of seed, and seed weight (g) of about 20 fruits were measured from each site. Fruit length (mm) and width (mm) were measured using a digital Vernier caliper (Mitutoyo, Japan) with 0.01 mm sensitivity. Fruit and seed weight were measured by digital weight balance (Mettler Toledo, Colombus, USA) having weight sensitivity up to 0.001 g.

### 2.5 Fruit chemical and biochemical properties

To study the chemical and biochemical properties of the fruits, we collected twenty fruits from each plant from each site. The total soluble solid contents and sugar contents were measured in Brix using a digital refractometer (Atago-Japan) for all fruit samples collected from all experimental fields (open and control pollination). The phenolic contents of fruit peel extracts were measured using the modified colorimetric Folin-Ciocalteu method (Zhang et al., 2015) and expressed as mg GAE/g DW (Gallic Acid Equivalent in dry plant material). Loquat fruit antioxidant capacity was estimated through three assays 2,2-Diphenyl-1-picrylhydrazyl radicals (DPPH), 2,2′-azino-bis (3-ethylbenzothiazoline-6-sulfonate (ABTS), and ferric reducing antioxidant power (FRAP) (Xu et al., 2014, Zhang et al., 2015). The moisture contents of the fruits were measured by the standard drying method (Saberian et al., 2014). The samples were subjected to the oven after preparation; the oven had a vacuum pump specific for drying. The formula used to determine the moisture content (Mc = total weight-dry weight/total weight × 100). Loquat juice titratable acidity was determined using NaOH 0.1 N and phenolphthalein as an indicator. It was expressed as % of malic acid and was calculated using the formula (% acid = (ml NaOH used) (0.1) (0.06)/(weight of sample) (10 ml)) (Hong et al., 2008, Garen et al., 2016). The flavonoids contents were measured by the aluminum calorimetric method (Quettier-Deleu et al., 2000, Meda et al., 2005), with rutin as a standard. Briefly, the samples were individually dissolved in dimethyl sulphoxide (DMSO). Then the sample solution was mixed with 150 of 2% aluminium trichlorde (AlCl_3_). After the incubation at ambient temperature for 10 min, the level of absorbance of the supernatant was measured at 435 nm using the spectrophotometer. The total dietary fiber was measured by using the enzymatic–gravimetric method according to Prosky et al. (1988).

### 2.6 Statistical analysis

All measurements on fruits were performed in triplicate, and results were presented in mean ± standard error (S. Error), all statistical data analyzed through statistical package SPSS (version 26). The effect of insect pollinators diversity on the fruit quality, physiological and chemical parameters were determined using an analysis of variance (ANOVA). Student’s *t*-test was used to test the significant difference between two groups, one-way ANOVA followed by Tukey post-hoc test was used to determine the difference between three or more groups. Differences between means at the 95% (p < 0.05) confidence level were considered statistically significant, while differences at the 99% (p < 0.01) confidence level were considered highly significant.

## 3 Results

### 3.1 Insect pollinators diversity and their relative abundance

As the Loquat tree starts flowering in late winter, it was noted that relatively limited numbers of pollinators were visiting its flowers during all the period from 2017 to 2019. All the insect pollinators recorded during observations of loquat trees were mention (Table 1**).** A total of 22 species from 3 families (Apidae, Halictidae, and Syrphidae) and 2 orders (Hymenoptera and Diptera) were collected and identified during the flowering season (Table 1). Most of the insects were belonging to the order Hymenoptera followed by Diptera. The bees were the most diverse group during the flowering season and corresponded to 14 species, while flies were represented by only 5 species (Table 1). The Apidae was the most frequently observed family represented (13 species) while the Halictidae was represented by one species. In the case of pollinator flies, only one family of Syrphidae was identified throughout the flowering season during the study period.

**Table 1:** Insect pollinators diversity on loquat tree during 2017–2019.

Order	Family	Pollinators	Pollinator species
Hymenoptera	Apidae	Honey bee	*Apis cerana*
			*Apis dorsata*
			*Apis mellifera*
			*Apis florea*
		Blue banded bees	*Amegilla niveocincta*
			*Amegilla insularis*
			*Amegilla confuse*
			*Amegilla cingulata*
			*Amegilla zonata*
		Cuckoo bees	*Thyreus ramosus*
		Carpenter bees	*Ceratina sexmaculata*
			*Ceratina binghami*
			*Xylocopa irridipennis*
		Bumble bee	*Bombus haemorrhoidalis*
			*Bombus asiaicus*
		Flower bees	*Anthophora pulcherrima*
	Halictidae	Sweat bees	*Nomia oxybeloiues*
Diptera	Syrphidae	Hoverfly	*Eristalis smilis*
			*Eupeodes corollae*
			*Eristalinus aeneus*
			*Ischiodon scutellaris*
			*Cheilosia albipila*

**Fig. 1:**
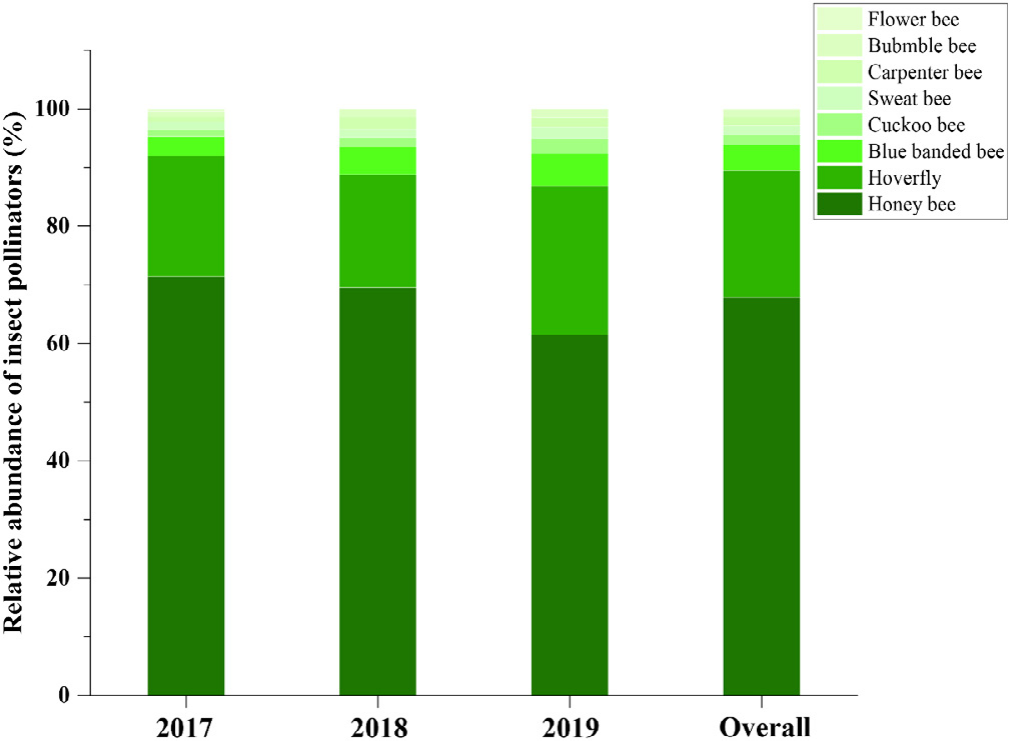
Percent relative abundance of insect pollinator visits by broad taxonomic group on loquat tree during 2017–2019.

The percent relative abundance of insect pollinators by major taxonomic groups on loquat trees during 2017–2019 is presented (Fig. 1). The major groups were honey bees (67.89%) and hoverflies (21.57%). Blue-banded bee and Cuckoo bees represented 4.43% and 1.75%, respectively. Other groups (sweet bees, carpenter bees, bumblebees, and flower bees) were less frequent.

**Fig. 2:**
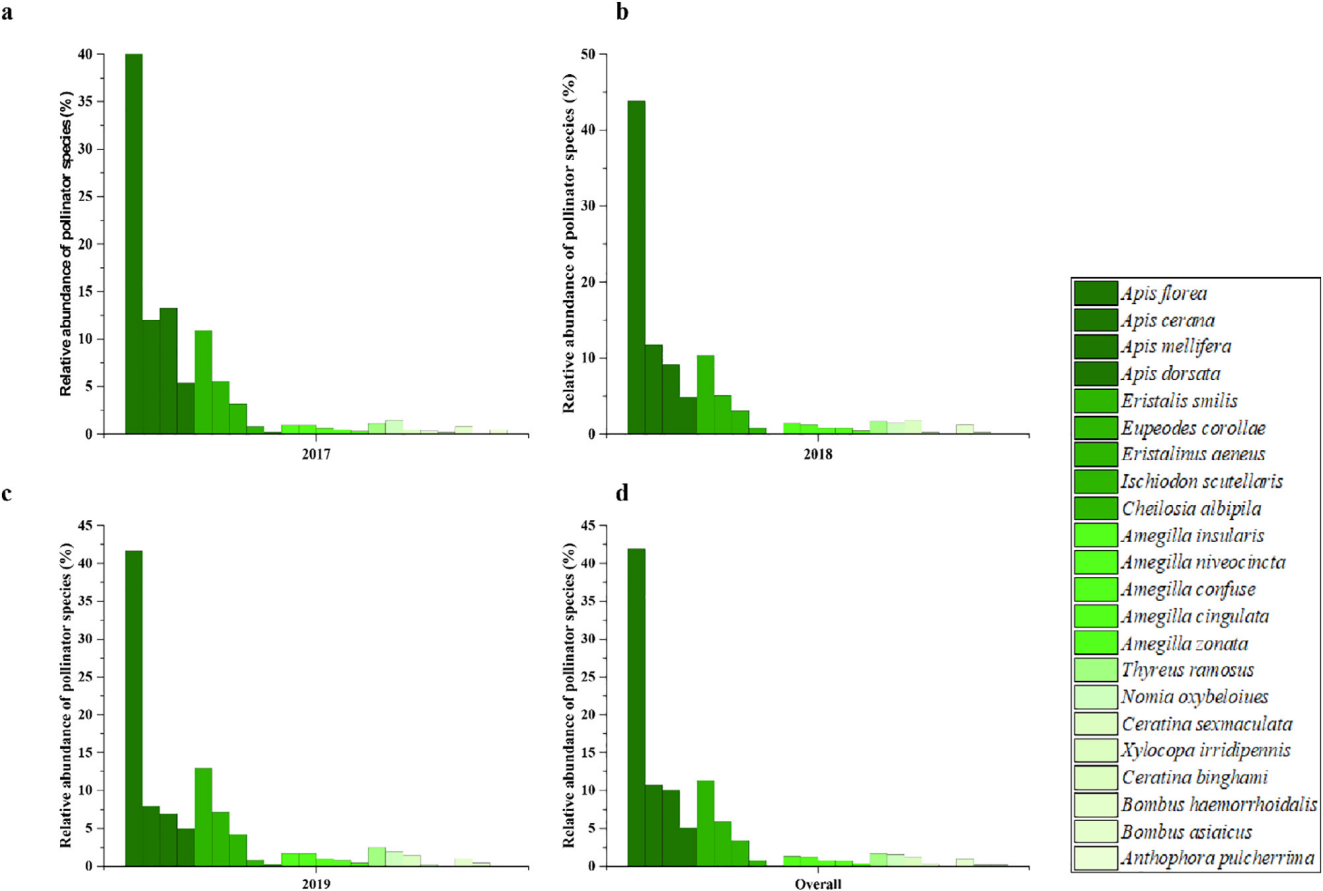
Percent relative abundance of insect pollinator species on loquat during 2017 (a), 2018 (b), 2019 (c), and overall (d).

The percent relative abundance of insect pollinator species on loquat trees during 2017–19 is described (Fig. 2). Overall results reveal that the percent relative abundance of honey bee species (*Apis florea* (41.96%), *Apis cerana* (10.72%), *Apis mellifera* (10.10%), *Apis dorsata* (5.11%)) is higher followed by hoverflies (*Eristalis smilis* (11.35%), *Eupeodes corollae* (5.86%)*, Eristalinus aeneus* (3.43%)*, Ischiodon scutellaris* (0.81%) *, Cheilosia albipila* (0.12%) during the flowering season of the loquat tree. While the less percent relative abundance was observed in bumblebee species (*Bombus haemorrhoidalis* (1.00%) *and Bombus asiaicus* (0.19%) *and Anthophora pulcherrima* (0.19%).

### 3.2 Fruit yield

Increases fruit yield per loquat tree in open-pollinated flowers as compared to control pollination is mention (Table 2). The results indicated that the fruit yield by the open pollination was significantly higher than the control pollination (t = 17.79; p = 0.001). Mean (±S. Error) fruit yield per tree was 22.31 ± 0.34 kg in open-pollinated flowers, whereas fruit yield in control pollination was 14.80 ± 0.25 kg/tree. The non-significant difference was present in fruit yield in open pollination during 2017–2019 (F (2,18) = 0.164, P = 0.850). Similarly, a non-significant difference was observed in fruit yield in control pollination during 2017–2019 (F (2,18) = 0.01, P = 0.990).

**Table 2:** Effect of open and control pollination on loquat yield (kg) per tree during 2017–2019.

Contents	Yield (kg)/tree			
	2017	2018	2019	Overall
	Mean ± S. Error	Mean ± S. Error	Mean ± S. Error	Mean ± S. Error
Open pollination	22.16 ± 0.72 a	22.59 ± 0.52 a	22.16 ± 0.59 a	22.31 ± 0.34 a
Control pollination	14.77 ± 0.44 a	14.84 ± 0.58 a	14.76 ± 0.32 a	14.80 ± 0.25b

### 3.3 Effect on fruit physiological characters

Physical parameters of fruits were changed significantly when compared to the fruit obtained from open-pollinated and control pollinated flowers (Table 3). Fruits weight differed significantly in open pollination as compared to control pollination (t = 21.22; p = 0.001). The fruit weight was (20.87 ± 0.24 g) in open pollination, while in control pollination was (13.68 ± 0.24 g). There was a non-significant difference in fruit weight was observed within open and control pollination. The size of fruit (mm) was significantly different in open pollination as compared to control pollination (t = 12.17; p = 0.001). The fruit size was (27.15 ± 0.23 mm) in open pollination, while in control pollination was (19.5 ± 0.59 mm). In the case of fruit diameter, there was a significant difference present in open pollination as compared to control pollination (t = 6.24; p = 0.001). The average diameter of fruit was (1.79 ± 0.02 cm) in open pollination, whereas the fruit diameter in control pollinated flowers was (1.45 ± 0.5 cm). Seed weight in loquat different significantly in open pollinators tree as compared to control pollinator tree (t = 15.34; p = 0.001) with heavier seeds obtained from open-pollinated flowers (1.87 ± 0.13 g) as compared to bee-pollinated flower (1.50 ± 0.21 g). Overall results indicated that there was a non-significant difference of physical parameters were observed within open and control pollination (Table 3).

**Table 3:** Effect of open and control pollination on physical parameters of fruit during 2017–2019.

Contents	Open pollination			Control pollination			Open pollination	Control pollination
	2017	2018	2019	2017	2018	2019	Overall	
	Mean ± S. Error	Mean ± S. Error	Mean ± S. Error	Mean ± S. Error	Mean ± S. Error	Mean ± S. Error	Mean ± S. Error	Mean ± S. Error
Fruit weight (g)	20.66 ± 0.37	20.71 ± 0.49	21.25 ± 0.42	13.94 ± 0.29	12.73 ± 0.23	14.39 ± 0.41	20.87 ± 0.24 a	13.68 ± 0.24b
Fruit Size (mm)	26.88 ± 0.46	27.78 ± 0.34	26.79 ± 0.31	19.99 ± 1.33	19.03 ± 0.93	19.48 ± 0.85	27.15 ± 0.23 a	19.5 ± 0.59b
Fruit diameter (cm)	1.78 ± 0.04	1.77 ± 0.02	1.83 ± 0.03	1.51 ± 0.10	1.40 ± 0.01	1.44 ± 0.07	1.79 ± 0.02 a	1.45 ± 0.5b
Seed weight (g)	1.88 ± 0.02	1.87 ± 0.03	1.88 ± 0.02	1.51 ± 0.03	1.49 ± 0.01	1.49 ± 0.06	1.87 ± 0.13 a	1.50 ± 0.21b

### 3.4 Effect of open and control pollination on chemical parameters of fruit

The results revealed that a significant difference was observed in the chemical parameters of fruit produced by open and control pollination during 2017–2019. The total sugar, phenolic content, antioxidant contents, total soluble solids, moisture contents, total organic acid, total flavonoids, and total dietary fiber contents values for loquat were mention (Table 4). The sugar content of loquat fruit is significantly more in the open-pollinated tree as compared to the control pollinated tree (t = 17.27; p = 0.001). The value of sugar content in open and control pollination was (6.23 ± 0.10 Brix) and (3.98 ± 0.08 Brix) respectively. Similarly, the phenolic content (mg TEAC/g DW) of loquat fruit differed significantly in open and control pollination (t = 8.67; p = 0.001) and their value was (0.62 ± 0.01) and (0.52 ± 0.01). Antioxidant content (mg TEAC/g DW) was significantly (t = 10.34; p = 0.001) higher in loquat fruit that is obtained from open-pollinated flowers (32.15 ± 0.62) as compared to control pollinated flowers (1.45 ± 0.5). The percentage of total soluble solid was significantly (t = 7.60; p = 0.001) more in open pollination (7.67 ± 0.16) than the control pollination (5.81 ± 0.18) of loquat fruit. Moisture content was significantly (t = 12.29; p = 0.001) more in loquat fruit that was obtained from the open-pollinated flower as compared to control pollinated flowers. The value of moisture contents (g 100 g^−1^) were (82.57 ± 0.35) and (71.36 ± 0.85), respectively. Further, the total organic acid contents TOS (mg 100 g^−1^ FW) differed significantly (t = 5.87; p = 0.001) in open-pollinated fruits (487.68 ± 2.44) than control pollinated fruits (402.86 ± 14). The amount of flavonoids contents within fruit was significantly higher (t = 9.43; p = 0.001) than obtained from open-pollinated flowers than control pollinated flowers. Similarly, total dietary fiber (g 100 g^−1^) within the open-pollinated fruits (1.19 ± 0.01) was significantly different (t = 13.47; p = 0.001) as compared to the fruits obtained from control pollinated flowers (1.14 ± 0.01).

**Table 4:** Effect of open and control pollination on chemical parameters of loquat fruit during 2017–2019.

Contents	Open pollination			Control pollination			Open pollination	Control pollination
	2017	2018	2019	2017	2018	2019	Overall	
	Mean ± S. Error	Mean ± S. Error	Mean ± S. Error	Mean ± S. Error	Mean ± S. Error	Mean ± S. Error	Mean ± S. Error	Mean ± S. Error
Sugar content (Brix)	6.27 ± 0.17	6.21 ± 0.17	6.24 ± 0.12	3.97 ± 0.11	13.95 ± 0.22	4.04 ± 0.06	6.23 ± 0.10 a	3.98 ± 0.08b
Phenolic contents (mg GAE/g DW)	0.62 ± 0.03	0.62 ± 0.02	0.62 ± 0.01	0.53 ± 0.01	0.53 ± 0.01	0.51 ± 0.01	0.62 ± 0.01 a	0.52 ± 0.01b
Anti-oxidant content (mg TEAC/g DW)	32.13 ± 0.99	32.01 ± 1.44	32.32 ± 0.90	22.87 ± 1.01	23.64 ± 0.62	23.63 ± 0.61	32.15 ± 0.62 a	23.28 ± 0.59b
Total Soluble solids (%)	7.74 ± 0.31	7.79 ± 0.29	7.48 ± 0.28	5.81 ± 0.32	5.88 ± 0.32	5.71 ± 0.40	7.67 ± 0.16 a	5.81 ± 0.18b
Moisture contents (g 100 g^−1^)	82.67 ± 0.76	82.25 ± 0.76	82.78 ± 0.25	71.52 ± 0.86	71.04 ± 2.36	71.53 ± 0.91	82.57 ± 0.35 a	71.36 ± 0.85b
Total organic acid contents TOS (mg 100 g^−1^ FW)	488.71 ± 2.21	487.37 ± 3.87	486.96 ± 6.29	403.13 ± 28.08	404.26 ± 25.61	401.2 ± 24.01	487.68 ± 2.44 a	402.86 ± 14.22b
Total flavonoids (mg Rutin/g FW)	0.08 ± 0.01	0.09 ± 0.01	0.09 ± 0.01	0.06 ± 0.01	0.06 ± 0.01	0.05 ± 0.01	0.09 ± 0.01 a	1.06 ± 0.01b
Total dietary fiber (g 100 g^−1^)	1.19 ± 0.01	1.19 ± 0.01	1.19 ± 0.01	1.14 ± 0.01	1.13 ± 0.01	1.14 ± 0.01	1.19 ± 0.01 a	1.14 ± 0.01b

In contrast, there was a non-significant difference of chemical parameters were observed within the fruit that was open-pollinated flowers and control pollinated flowers (Table 4).

## 4 Discussion

Even though loquat is considered a self-compatible fruit species, its flowers have a high amount of nectar and pollen for the pollinator insects (McGregor, 1976). In the present study, 22 pollinator species belonging to three families (Apidae, Syrphidae, and Halictidae) were collected on a loquat orchard. Hymenopteran was the most dominant order on the loquat orchard. In contrast, Sarwar et al. (2012) reported that the Dipterans were most abundant group of pollinators in loquat orchards. Our results indicated that the relative abundance of honey bee species (*Apis florea, Apis cerana*, *Apis mellifera*, *Apis dorsata*) and hoverfly species (*Eristalis smilis*, *Eupeodes corollae, Eristalinus aeneus, Ischiodon scutellaris, Cheilosia albipila*) being two major groups.

Our results revealed that the physiological and chemical parameters of the loquat fruit were strongly linked to insect pollinators. The effect of pollination on the loquat fruit physical characteristics was already reported (Freihat et al., 2008). Freihat et al. (2008) demonstrated the impact of different pollination methods on the fruit set and quality of loquat (Crane and Caldeira, 2006). The pollinators cut off strongly affected the fruit size and weight as compared to open pollination (Garratt et al., 2014, Abrol et al., 2019). Results also proved that loquat fruit qualities were strongly linked to the population and type of pollinators. The lowest physiological and chemical characters were obtained in case of controlled conditions as compared to open pollination. In controlled pollination where the honey bees (*Apis mellifera*) were used as the main pollinator, consistent but low physical and chemical qualities fruit yield and fruit set percentage were recorded throughout the study period. Our results confirmed a previous study on loquat fruit (Sarwar et al., 2012) and some other fruits as mangoes (Rafique et al., 2016), and strawberries (Garratt et al., 2014, Abrol et al., 2019). Sarwar et al. (2012) also found that the mean weight (g) of individual fruits was significantly higher that obtained from the open-pollinated flowers as compared to un-pollinated flowers. Low temperature and the rainfall during the blossoming period also negatively affected the pollination at all study sites and open pollination.

The sugar contents and antioxidant properties were the two most affected characters by the pollination. These results supported the studies conducted on apples and other fruits (Mahmood et al., 2017, Abrol et al., 2019). Our result revealed that the total phenolic contents of the fruit peel differed significantly from that obtained from the open pollination or control pollinated flowers. However, the amount of total phenolic contents is varied that have been present in loquat cultivars in China (Zhang et al., 2015), Japan (Ding et al., 2001), Turkey (Ercisli et al., 2012), and America (Pande and Akoh, 2010). These variations in phenolic contents of loquat fruits due to environmental and genetic factors during before and after harvesting conditions. The fruit production quantity, morphology, and chemical properties were observed lowest throughout the study in controlled conditions. Flesh weight, thickness, and sugar contents were negatively related to controlled pollination. Similarly, the studies conducted in Spain and Jordan revealed that the fruits from open-pollinated flowers had better qualities than those issued from controlled pollination (Freihat et al., 2008). The variation of physiological and chemical parameters from others studies is due the type of variety, stage of majority, and others environmental factors.

Results indicated that the total yield, fruit set percentage, and other physical and chemical properties decrease rapidly with the decrease of pollinators diversity. The pollinators loss could be attributed to many contributing factors like pesticides, diseases, air pollution, climate change, monoculture, habitat loss, and land conversion as reported in previous studies (Potts et al., 2010, Winfree et al., 2011, Ahmad and Aziz, 2017, Al-Ghamdi et al., 2020, Mubeen et al., 2021). This not only affected the overall pollinator fauna of the area but also the quality and quantity of the fruit deteriorated very rapidly. Further study is needed to determine the effect of pollinators declining on physiological and chemical parameters of loquat fruits.

## 5 Conclusions

Loquat fruit is one of the significant sources of income for the farmer community of the Pothwar region of Pakistan. This is the first time; a field study has been undertaken to determine the effect of insect pollinators richness and their abundance on the production quantity and the qualitative parameters of loquat fruits. The results suggest that native insect pollinator communities play a significant role to increase fruit quality and production. Most of the physiochemical parameters of fruits are directly linked to the pollinator diversity as well as the pollination technique. Therefore, it is necessary to maintain the habitat of insect pollinators near loquat orchards to ensure good productivity and fruit quality.

## Declaration of Competing Interest

All authors declare that they have no known competing financial interests or personal relations that could have appeared to influence the work reported in this paper.
